# Penile refracture: a preliminary report

**DOI:** 10.1590/S1677-5538.IBJU.2018.0124

**Published:** 2018

**Authors:** Rodrigo Barros, Matheus Guimarães, César Nascimento, Luis Rogério Araújo, Leandro Koifman, Luciano Alves Favorito

**Affiliations:** 1 Hospital Municipal Souza Aguiar, Rio de Janeiro, RJ, Brasil; 2 Universidade do Estado do Rio de Janeiro, UERJ, Rio de Janeiro, RJ, Brasil; 3 Serviço de Urologia do Hospital Federal da Lagoa, Rio de Janeiro, RJ, Brasil

**Keywords:** Penis, etiology [Subheading], Diagnostic Techniques, Surgical

## Abstract

**Objective:**

To report our institutional experience with penile refracture, including demographic data, recurrence time, etiology and operative findings in the first and second episodes.

**Materials and methods:**

Between January 1982 and September 2017, 281 patients underwent surgical treatment for penile fracture (PF) at our institution. Demographic data, clinical presentation, besides operative findings and follow-up of patients with relapsed PF were retrospectively assessed by reviewing medical records.

**Results:**

Of a total of 281 cases of PF operated at our institution, 3 (1.06%) patients experienced two episodes of trauma. Age ranged from 38 – 40 years (mean: 39.3). The recurrence time varied from 45 to 1560 days (mean: 705). Two patients presented the new fracture episode at the same site of the previous lesion, while in the other case the lesion was observed at another site.

**Conclusion:**

Recurrent FP is an extremely rare entity. The risk factors for its occurrence are still unknown. Although the lesion of the corpus cavernosum ipsilateral to the scar tissue of the prior FP is more common, contralateral rupture may be present. Nevertheless, prospective studies with larger samples should be conducted.

## INTRODUCTION

Penile fracture (PF) represents a rare urologic emergency situation, corresponding to 1 in every 175.000 emergency hospital visits (1). A recent literature review evaluating data from different regions of Iran has estimated that the incidence of PF in the Middle Eastern country can be estimated to be between 1.1 and 9.9 per 100.000 male inhabitants, being that urologists encounter, on average, 1 patient with FP in every 3.5 months (2). However, this is probably an underreported entity, due to the possible shame of patients seeking medical attention. The actual incidence of PF is possibly much higher than that reported in the literature (3).

The occurrence of a second episode of PF consists of an even rare situation, with only 10 cases described in the world literature (4–13). The aim of this study is to evaluate the demographic data, recurrence time, etiology and operative findings in the first and second episodes.

## MATERIALS AND METHODS

Between January 1982 and November 2017, 281 patients underwent surgical treatment for PF at our institution. Demographic data, etiology, clinical presentation and operative findings of patients with penile refracture, besides recurrence time between first and second episodes were retrospectively assessed by reviewing medical records. The injury mechanism and the sexual position were assessed.

All patients underwent the standardized surgical technique in our institution, as previously described (4), which consists of penile degloving through subcoronal incision. In this access, lesions of the corpora cavernosa are identified and the tunica albuginea is sutured with separate stitches of 3 - 0 Polyglactin. Associated partial urethral lesions are treated primarily through simple suturing with 5 - 0 Polyglactin. Postectomy is routinely performed in all uncircumcised patients. Bilateral rupture of the CC, with or without associated urethral transection were classified as severe. The patients were evaluated after six months follow-up.

The experimental protocol described below was approved by the ethical committee for human experimentation of our university, and the study was carried out in accordance with the ethical standards of the hospital's institutional committee on human experimentation.

## RESULTS

Of a total of 281 cases of PF operated at our institution, 3 (1.06%) patients experienced a second episode of PF. The age, etiology of the first and second episode, the recurrence time and the type of the two fractures of the 3 patients with penile refracture can be observed in Table-1.

**Table 1:** The table shows the demographic data and operative findings of the 3 cases of penile refracture in our sample.

Patient	Age	Etiology (First episode)	Etiology (Second episode)	Recurrence time	Type of lesion (First episode)	Type of lesion (Second episode)
1	38	Anal intercourse/“doggy style” position	Anal intercourse/“doggy style” position	52 months	Right CC (distal portion)	Right CC (distal portion)
2	40	Anal intercourse/“man-on-top” position	Vaginal intercourse/“doggy style” position	45 days	Right CC (proximal portion)	Bilateral CC (medial shaft)
3	40	Refused	Refused	17 months	Left CC (distal portion)	Left CC (distal portion)

**CC** = corpus cavernosum

**Figure 1:**
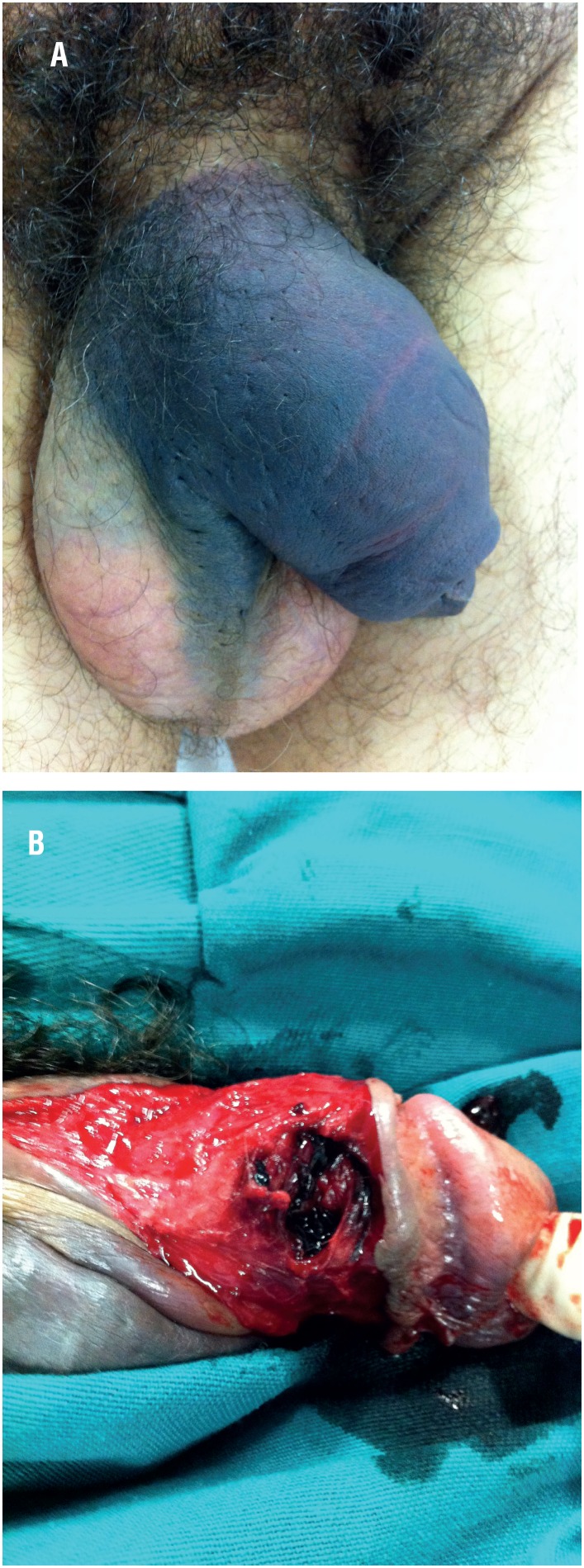
The figure shows a 38 - year - old patient with penile fracture. A) we can observe the penile hematoma after the penile trauma. B) Surgical exploration revealed injury to the distal portion of the right corpus cavernosum.

Case 1: 38-year-old, white, heterosexual patient entered our emergency room in June 2016 with pain and penile hematoma 19 hours after trauma during anal intercourse with his wife, who was in the “doggy style” position. Surgical exploration revealed injury to the distal portion of the right corpus cavernosum (Figure-1). The patient underwent surgical reconstruction with satisfactory evolution. This same patient underwent surgical treatment for a second FP in our department 52 months ago. The chart review disclosed that he was operated 33 hours after trauma during anal intercourse with his wife in “doggy style” position. During surgery, an injury was observed in the distal portion of the right corpus cavernosum, as well as in the first episode. After 6 months of the refracture, the patient progressed satisfactorily, without any sexual complaints.

**Figure 2:**
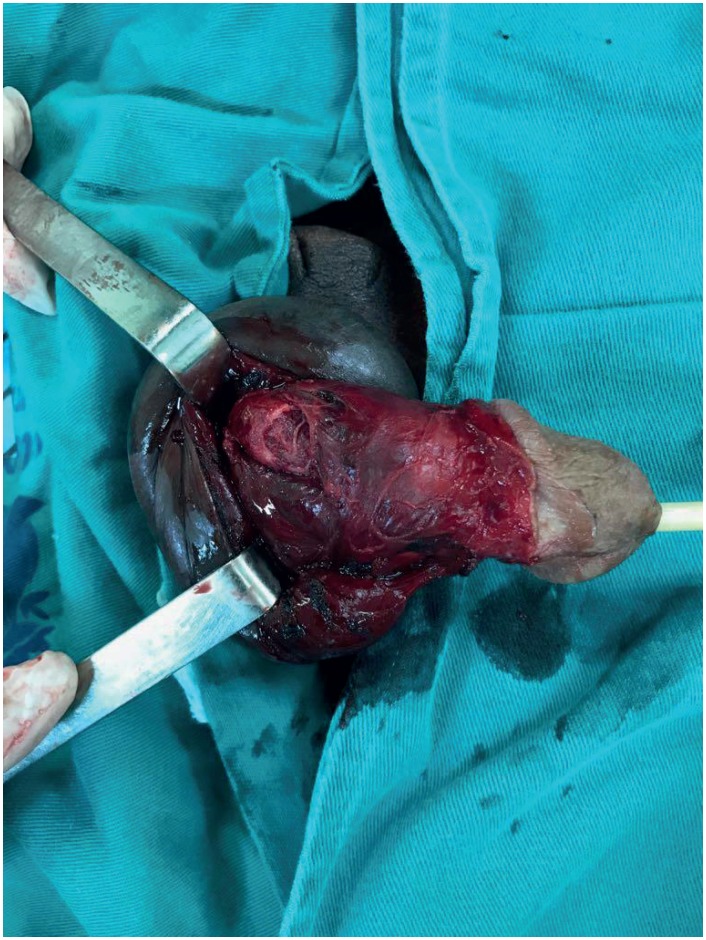
The figure shows a 40 - year - old patient with penile fracture. we can observe the lesion in the proximal segment of the right corpus cavernosum.

Case 2: A 40-year-old black heterosexual patient was admitted to our hospital in June 2013 with penile pain, immediate detumescence and eggplant deformity 3 hours after trauma during sexual intercourse with vaginal intercourse, and the wife in “doggy style” position. Surgical exploration demonstrated bilateral lesion of the corpora cavernosa in its medial shaft. Approximately 45 days earlier, this patient had undergone surgery for PF in our facility 14 hours after trauma during anal intercourse with “man - on - top” position. On that occasion, lesion was observed in the proximal segment of the right corpus cavernosum (Figure-2). This patient evolved with premature ejaculation, controlled after treatment with paroxetine.

Case 3: A 40-year-old black patient sought care in our hospital in December 2014 with pain, cracking and penile hematoma suggestive of PF, with 3 hours of evolution. He refused to provide data on the etiology of the trauma, but revealed that he had been operated on by us for the same reason 17 months ago. He was submitted to immediate surgical exploration, in which an injury was observed in the distal portion of the left corpus cavernosum. The patient's chart review disclosed that he had been operated on for FP 9 hours after trauma, not providing details, with injury finding at the same site of the second fracture episode. This patient did not follow-up in our postoperative clinic.

## DISCUSSION

PF consists of a rare urologic emergency. It is believed that its incidence is much higher than that reported in the literature, since a large number of patients does not seek emergency medical care in virtue of embarrassment (14). This was demonstrated in our sample, where one of our patients, despite seeking medical attention, refused to provide personal information and details of the etiology of the trauma. Recurrent FP is an even rarer entity and no case has been documented in the main publications with higher casuistic (15). To date, only 10 cases have been reported in the world literature (4–11). In our knowledge, although small, this is the largest series of recurrent PF described in the literature to date.

The risk factors for recurrent fracture are difficult to verify due to their extreme rarity. However, some associations have been postulated. De Rose et al. (16) revealed histological evidence of an underlying chronic inflammatory process in the tunica albuginea of patients with PF. The fibrous and inelastic scar tissue of the anterior lesion seems to weaken the corpora cavernosa, making it weaker and vulnerable to a new fracture episode. This theory is supported by the predominance of PF cases that recur in the ipsilateral cavernous body (5, 10). In contrast, according to Sharma et al. (12), the scar tissue can lead to an unequal distribution of tension in the tunica albuginea, causing rupture of the contralateral side.

Although there is no standardized period of sexual abstinence to be recommended for patients operated on as a result of PF, most authors advice at least six weeks, which corresponds to the time at which collagen deposition is completed. El-Assmy et al. (17), as well as Kozacioglu et al. (18) instructed their patients to maintain abstinence for 6 weeks after PF surgery. In the study by Özorak et al. (19), it is evidenced that patients were instructed to abstain from sexual activity during the first 8 weeks after the surgical intervention. However, according to Prasanna et al. (20), ipsilateral recurrence is more likely to occur within two years after repair of the primary fracture. We routinely advise patients operated on the possibility of a refracture and all patients in our study were instructed to avoid intercourse for at least 8 weeks. However, one of our cases presented the second episode after only 45 days. Interestingly, in addition to injury at the same point of the primary repair, contralateral involvement was observed. This can be explained, in addition to the histological changes, by the fact that the “doggy - style” position is generally associated with more severe lesions, with bilateral involvement of the corpora cavernosa and urethra (21).

Some authors recommend the use of non - absorbable suture material in PF repair to minimize the risk of recurrence. Nonetheless, there is no evidence to suggest that nonabsorbable material would result in less fracture recurrence. In addition, knots can be felt under the thin skin of the penis, which can cause discomfort during sexual intercourse (5).

Ridyard et al. (11) reported a case of relapsed PF in which the patient was under the influence of alcohol at the time of both episodes of trauma and raised the hypothesis that drug or alcohol abuse may predispose to this type of injury. However, we did not observe this association in any of our cases.

Traumatic experience with PF may raise fears about upcoming sexual intercourse, leading to performance anxiety and the development of ejaculatory dysfunctions (22). One of the patients in our study developed secondary premature ejaculation after recurrence of PF.

## CONCLUSIONS

Recurrent PF is an extremely rare entity. However, patients should be advised of this possibility after the first episode of PF. The risk factors for its occurrence are still unknown. Although the lesion of the corpus cavernosum ipsilateral to the scar tissue of the prior FP is more common, contralateral rupture may be present. Nevertheless, prospective studies with larger samples should be conducted.
